# Computational tradeoffs in multiplex PCR assay design for SNP genotyping

**DOI:** 10.1186/1471-2164-6-102

**Published:** 2005-07-25

**Authors:** John Rachlin, Chunming Ding, Charles Cantor, Simon Kasif

**Affiliations:** 1 Bioinformatics program, Boston University, Boston MA 02215, USA; 2 Centre for Emerging Infectious Diseases, The Chinese University of Hong Kong, Prince of Wales Hospital, Shatin, New Territories, Hong Kong Special Administrative Region, Hong Kong; 3 Department of Biomedical Engineering, Boston University, MA 02215, USA; 4 Center for Advanced Biotechnology, Boston University, MA 02215, USA; 5 Center for Advanced Genomic Technologies, Boston University, MA 02215, USA; 6 SEQUENOM, Inc., San Diego, CA 92121-1331, USA

## Abstract

**Background:**

Multiplex PCR is a key technology for detecting infectious microorganisms, whole-genome sequencing, forensic analysis, and for enabling flexible yet low-cost genotyping. However, the design of a multiplex PCR assays requires the consideration of multiple competing objectives and physical constraints, and extensive computational analysis must be performed in order to identify the possible formation of primer-dimers that can negatively impact product yield.

**Results:**

This paper examines the computational design limits of multiplex PCR in the context of SNP genotyping and examines tradeoffs associated with several key design factors including multiplexing level (the number of primer pairs per tube), coverage (the % of SNP whose associated primers are actually assigned to one of several available tube), and tube-size uniformity. We also examine how design performance depends on the total number of available SNPs from which to choose, and primer stringency criterial. We show that finding high-multiplexing/high-coverage designs is subject to a computational phase transition, becoming dramatically more difficult when the probability of primer pair interaction exceeds a critical threshold. The precise location of this critical transition point depends on the number of available SNPs and the level of multiplexing required. We also demonstrate how coverage performance is impacted by the number of available snps, primer selection criteria, and target multiplexing levels.

**Conclusion:**

The presence of a phase transition suggests limits to scaling Multiplex PCR performance for high-throughput genomics applications. Achieving broad SNP coverage rapidly transitions from being very easy to very hard as the target multiplexing level (# of primer pairs per tube) increases. The onset of a phase transition can be "delayed" by having a larger pool of SNPs, or loosening primer selection constraints so as to increase the number of candidate primer pairs per SNP, though the latter may produce other adverse effects. The resulting design performance tradeoffs define a benchmark that can serve as the basis for comparing competing multiplex PCR design optimization algorithms and can also provide general rules-of-thumb to experimentalists seeking to understand the performance limits of standard multiplex PCR.

## Background

The PCR (Polymerase Chain Reaction) method of DNA amplification has had a profound impact on biotechnology and biological research. Multiplex PCR is an extension of the standard PCR protocol in which multiple loci are amplified simultaneously in order to save time, improve throughput, and reduce the total cost of reagents. Applications for PCR and Multiplex PCR abound including quantitative gene expression [1-4], haplotyping [5], whole-genome closure [6,7], detection of genetically modified organisms [8], forensic analysis, including human identification and paternity testing [9,10] diagnosis of infectious diseases [11,12], and for anti-bioterror applications aimed at detecting biological agents such as Anthrax [13]

Multiplex PCR has recently emerged as a core enabling technology for high-throughput SNP genotyping [14-16], and variations on the standard protocol are being actively explored and in some cases more widely commercialized. It is in this context of genotyping that we focus our discussion of multiplex PCR assay design. Thus we will typically refer to multiplexing SNPs (rather than primers) but our treatment is readily applicable to most other PCR applications. Genomic variations in the form of Single Nucleotide Polymorphisms (SNPs) and associated haplotypes continue to garner tremendous interest particularly in the context of pharmacogenomic initiatives aimed at understanding the connection between individual genetic traits, drug response, and disease susceptibility [17-21]. Broad adaptation of genotyping technologies in clinical settings will depend on their cost and inherent clinical value and may be significantly impacted by ethical and legal considerations. Recent technological developments in PCR-based genotyping based on primer extension with universal PCR primers [22] have demonstrated very high (~100-plex) multiplexing levels, although the use of common primers does introduce other issues including the greater potential for cross-contamination.

Multiplex PCR assay design is a multi-objective optimization problem involving intrinsic performance tradeoffs. The key objectives we consider in this paper include the number of SNPs per tube (multiplex level) and the percentage of SNPs assigned to full tubes (coverage). We further require that all resulting tubes achieve uniform levels of multiplexing with the idea that doing so facilitates automation in a high-throughput environment. While lower coverage may be acceptable in initial survey studies involving many (10^4^-10^6^) SNPs, achieving high (>95%) coverage becomes obviously more important when the focus of investigation has been narrowed to a relatively small (10^2^-10^3^) set of SNPs each of which is suspected of having some biological or pharmacological impact.

The question we address in this paper is whether there are fundamental limitations to our ability to design assays that achieve multiplexing levels of arbitrary size using standard multiplex PCR protocols. While multiplex PCR is an established technique, its usefulness as the basis for future high-throughput platforms depends critically on scalability. We introduce a new framework of "multi-node graphs" to model the multiplex PCR problem. We show that the problem of finding high-multiplexing/high-coverage designs is subject to a computational phase transition, becoming dramatically more difficult when the probability that two primers are mutually compatible drops below a critical threshold. This probability depends on fundamental primer selection criteria typically selected to avoid the formation of primer dimers. For standard criteria, we can identify where such a transition occurs, and show that it is consistent with typical multiplexing levels. The precise location of this critical transition point will also depend on N, the number of available SNPs. For a given level of coverage, the level of achievable multiplex is proportional to log(N). We further quantify design performance tradeoffs using two best-fit tube assignment algorithms on human SNP data.

## Results

### Phase transitions in multiplex PCR complexity

Our first main result reported in this section can be succinctly stated as follows: for an assay with N SNPs and approximately S candidate primers for each SNP we devise a relatively efficient algorithm that can achieve almost perfect coverage with tubes of size approximately **O**(log NS). Unfortunately the coverage drops dramatically if the multiplex level is increased. (See [23] for a formal analysis.) This result is similar in spirit (but not in detail) to related observations made about other graph problems.

**Figure 1:**
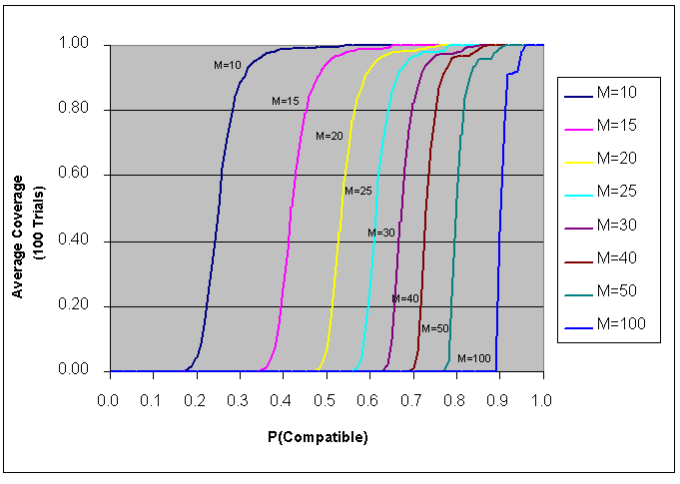
**Phase transition in full-tube coverage as a function of SNP-SNP compatibility probability. **These results are based on a simulations where the controlling parameter P denotes the probability that two SNPs are compatible. Two SNPs are compatible if their associated primers are all pair-wise compatible. This simulation is based on N = 1,200 SNPS and S = 500 primer pairs per SNP. In reality, this compatibility probability, P, depends on the stringency by which primer pairs are tested for cross-interactions. As we increase the target multiplexing level, higher compatibility, beyond what are normally obtained using standard primer selection criteria is required, suggesting fundamental barriers to increasing target multiplexing levels.

In recent years, it has been shown that for broad classes of computationally intractable problems, there exist certain phase-transition boundaries across which the nature of the solutions and the computational effort needed to identify a solution changes dramatically [24]. When attempting to design multiplex PCR assays with high coverage, we observe a similar computational behavior on simulations using a novel graph formulation we call a multi-node graph (see Methods section). This graph representation consists of nodes representing SNPs and edges connecting two multiplex-compatible SNPs. Two SNPs are multiplex compatible if none of their associated primers interact. To model the fact that SNP compatibility depends on the assigned primers, we allow for the presence of an edge matrix E_uv_. In a multi-node graph, E_uv_[i][j] = 1 when node u with primer set i is compatible to node v with primer set j. We induced a random multi-node graph by setting E_uv_[i][j] = 1 with probability P for all node pairs u and v, in states i and j respectively. Using a simple greedy algorithm (see *Methods *section) we find that our ability to achieve high (>95%) coverage for randomly generated multi-node graphs critically depends on the compatibility probability, P, (or conversely the interaction probability (1-P)) as well as the target level of multiplexing. These results are presented in Figure 1. By sampling from chromosome 21 of the human genome, the actual probability that two SNPs are compatible is approximately 0.299. Figure 1 would suggest, therefore, that designing 10-plex assays from N = 1,200 SNPs is generally straightforward, but that increasing multiplex performance to 15- to 20-plex or beyond becomes extremely problematic. This appears to be consistent with current design practice though we emphasize that the location of the phase transition depends on both the total number of SNPs and the number of candidate primer pairs per SNP. We recognize, furthermore, that random multi-node graphs only approximately model the multiplex assay design problem because primer pair candidates derived from real sequence data are not truly independent. For example, primer pairs may share a forward or reverse primer, or they may significantly overlap. In addition, in the process of assay design optimization, primers within a single tube may take on certain sequence characteristics (e.g., high GT / GA / CT / CA content) that are intrinsically less likely to interact, and thus make higher-than-expected coverage possible for a given multiplexing target.

### Multiplex PCR performance on human SNP data

Next we implemented two multiplex PCR assay design algorithms and applied them to real SNP data obtained from the dbSNP database. We prescreened the 84,393 chromosome 21 SNPs contained in build 116 of dbSNP [25] for class 1 SNPs (strict single nucleotide polymorphisms) containing at least 200 bases of non-low-complexity sequence both upstream and downstream from the target SNP. This reduced our working set to 18,498 SNPs, 21.9% of the original total, from which 1,200 SNPs were randomly selected for experimental purposes. The GC content of the 401-base flanking sequence surrounding (including the SNPs themselves) was 41.9% +/- 11.0% in line with a 41% GC content for chromosome 21 and for the human genome as a whole [26].

**Figure 2:**
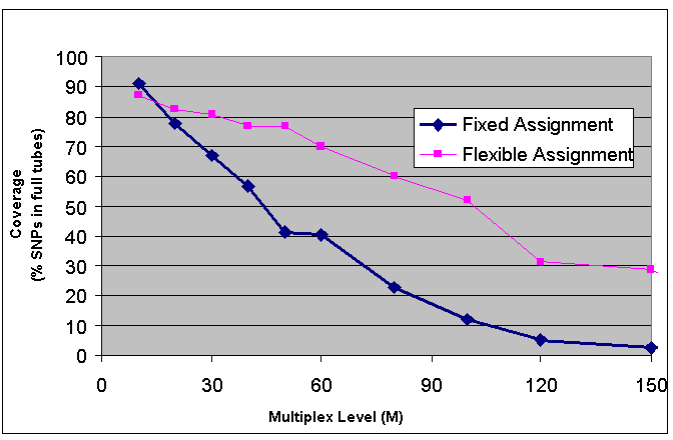
**Coverage vs. target multiplex level using two different best-fit tube assignment strategies. **These results were all based on N = 1,200 for varying target multiplexing level M. In each trial, the number of allowed tubes is limited to . Full-tube coverage, the percentage of SNPs assigned to full tubes, of close to 80% is achieved at a multiplexing level of 20, though it drops rapidly for higher multiplexing levels. The graph shows a significant improvement in one algorithm over the other, demonstrating that such tradeoffs can be used to effectively compare and contrast competing optimization strategies.

Our two best-fit greedy algorithms are designed to simultaneously assign primer candidates to SNPs and to partition SNPs into individual tubes in an effort to maximize both multiplexing level and coverage. See *Methods *for complete details. While best fit algorithms are relatively simple, one can actually show theoretically that these results appear to be as good as expected (on average) in graphs with this level of density. One version which we call "Fixed-Assignment Best Fit" assigns SNPs in random order to the fullest compatible tube, and as the name suggests, once a SNP is assigned to a tube it is fixed. Neither its assigned tube nor its associated primers are ever modified. If no compatible tube can be found, the SNP is left unassigned, reducing total coverage. We considered a second variation on the best-fit approach called "Flexible Assignment Best Fit" in which SNPs already assigned to a tube can be removed under special conditions in order to accommodate a new SNP assignment. Special rules of the algorithm guarantee that the algorithm will eventually terminate with increasingly high probability. Figure 2 demonstrates the precise nature of the tradeoff between multiplexing and SNP coverage for a fixed number of SNPs (N = 1,200) for both best fit methods.

### Multiplex PCR coverage performance tradeoffs

**Figure 3:**
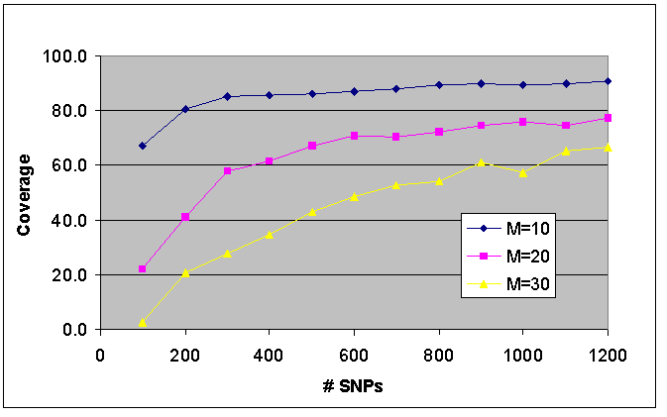
**Multiplex PCR performance tradeoffs. **A closer examination of the Fixed Assignment Best-Fit algorithm reveals tradeoffs between the available number of SNPs, N, the target multiplexing level, M, and full-tube coverage. The dip at N = 1000, M = 30 is an artifact of the algorithm which strictly limits the number of tubes to  = 34 tubes. Since M does not divide N evenly, the algorithm ends up partially filling the excess tube rather than working harder to fill the remaining 33 tubes to full 30-plex capacity.

Next we employed the fixed-assignment best-fit algorithm to generate coverage curves for target multiplexing levels M = 10, 20, 30 while varying numbers of SNPs. We considered SNP sets containing between 100 and 1200 SNPs. Figure 3 presents our results. With 200 SNPs, 80% coverage could be achieved with 10-plex assays, but this drops to 40% coverage using 20-plex assays. However, if we increase the number of SNPs to 1200, then for 20-plex assays, coverage increases from approximately 40% to 80%. This graph shows that regardless of the multiplexing level desired, coverage increases with the number of SNPs but with diminishing returns. More precisely, for fixed multiplexing level M, coverage is roughly proportional to log(N).

## Discussion

There is extensive literature on the general principles of PCR primer design [27-31]. This work has led to a number of software applications, most notably Primer3 and various extensions [32-34]. A fast dynamic programming formulation for testing primers for pair-wise compatibility has also been developed [35].

The application of Multiplex PCR has increased steadily over the past decade, requiring more sophisticated primer selection protocols. Different algorithms may favor particular objectives, or may be designed for particular technology platforms. In general, the problem of identifying primer pairs to maximize the multiplexing level of a single assay has been shown to be NP-complete by Nicodeme and Steyaert [36] who also present an approximation algorithm that eliminates 3' base complementarity while addressing product size constraints. They also consider electrophoresis distance constraints that require two amplicons to have some minimum length difference so that they can be distinguished after being processed through an electrophoresis gel. Additionally, SNP detection methods based on primer extension protocols in conjunction with mass spectrometry must take into account the resolution of the mass spectrometer as for example with the matching algorithms presented by Aumann, Manisterski, and Yakhini [37].

Whereas this paper focuses on the dual problem of assigning primers and partitioning SNPs into multiplex-compatible tubes, an entirely different multiplex PCR problem is concerned with finding a minimal number of primers necessary to amplify a maximum number of targets over a single experiment [38] or over multiple experiments [39].

Our best fit approach is motivated by the theoretical analysis provided by Alon and Furedi [40] who show that a greedy algorithm in standard graphs produces an independent set of size log N, and moreover this approach can be extended to produce a full cover of the graph. The multi-node graph is, in practice, substantially more complex to cover, however theoretical analysis suggests that the behavior is similar to standard graphs. The sketch of the proof is as follows. Formal details are provided elsewhere [23].

1. For a multi-node graph with N nodes and S states per node, we create a corresponding standard graph with NS nodes. (Each state in the multi-node graph is a unique node.)

2. We add random edges with probability P getting O(N^2 ^S^2 ^P) edges. Then we remove all the edges between nodes that are connecting representatives of the same node. The total number of edges removed is N S^2 ^P. This means independently of the number S of representatives per node we remove roughly 1/N of the total number of edges.

3. If 1/N << P then this removal does not greatly effect the resulting graph and the probability that their exists a clique of size K on a graph of size NS applies to a multi-node graph of size N with S representatives per node.

## Conclusion

In this paper, we quantified some of the critical tradeoffs involved in the multi-objective design of multiplex PCR assays and demonstrated a phase transition suggesting that achieving high-coverage designs becomes dramatically more difficult when SNP compatibility probabilities fall below a certain critical threshold. Explicit consideration of tradeoffs in multiplex PCR design is useful in helping researchers to design effective and reliable assays within the computational limits of the problem. Furthermore, such tradeoffs provide a natural basis for comparing and contrasting novel computational techniques aimed at improving one or more objectives. Although we have attempted to rely on standard design criteria, further laboratory testing is required to validate the design criteria used as the basis of this analysis. In the future we will seek to further improve our current tradeoff benchmarks with the development of novel algorithms and to apply our techniques to the design of high-multiplexing assays that achieve broad coverage of the complete human genome. We have also developed a web-enabled Multiplex PCR assay design system known as MuPlex [41] that also serves as a testing ground for on-going algorithmic development.

## Methods

### Multi-node graphs: a novel formulation for the multiplex PCR problem

Designing one or more multiplex assays for SNPs with preselected primers is equivalent to finding a clique in a graph G where nodes are SNPS and edges connect pairwise multiplex compatible SNPs, i.e., two SNPs whose primers can be pooled with a single tube without forming primer dimers. Equivalently, we can model the problem using the inverse graph  whose edges denote non-compatible or interacting SNPs (i.e., SNPs whose associated primers incur at least one interaction) in which case the objective is to identify an independent set rather than a clique. Theoretical bounds for covering a graph with disjoint cliques can be found in [42].

**Figure 4:**
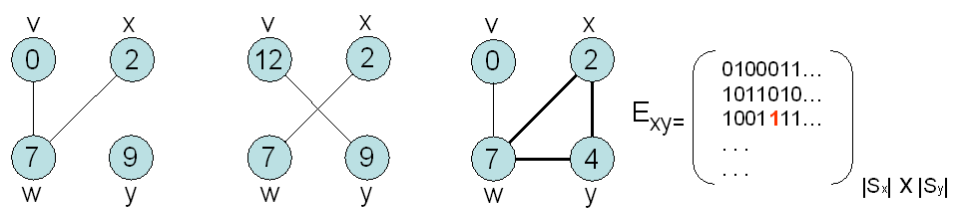
**Multi-node graphs. **A multi-node graph is a convenient way of formalizing the multiplex PCR problem. In multi-node graphs, individual nodes can take on one or more states. In this figure, an edge between two nodes, X and Y, is determined by the state of the two nodes, or more specifically, an edge matrix **E**_XY _connecting nodes X and Y. There is no restriction on the number of states per node, and each node may contain a different number of states.

The multiplex PCR problem is more general in that we impact the graph topology by choice of primers. We use the term "multi-node graph" to denote a graph whose nodes have multiple states. In a multi-node graph, an edge matrix E_uv _is attached to each pair of nodes, (u, v). If node u (in state i) is multiplex compatible with node v (in state j) then E_uv_[i][j]= 1. Otherwise, E_uv_[i][j] = 0. As illustrated in Figure 4, nodes W, X, and Y are pair-wise compatible when in certain states and incompatible in other states. The nodes W, X, Y form a 3-clique (3-plex) when in states 7, 2, and 4 respectively. The multiplex PCR design problem is equivalent to choosing a state assignment to each node in a graph to achieve maximal covering (including as many nodes as possible) with disjoint cliques of size M in a multi-node graph.

In the context of multiplex PCR, our goal is to identify a set of uniformly sized disjoint cliques in a multi-node graph. This involves the dual problem of selecting node states (primer pair selections for each SNP) and identification of the cliques themselves corresponding to multiplex-compatible SNP sets.

### Selecting candidate primers

We generated candidate forward and reverse primer pair candidates for each of the 1,200 SNPs according to the selection criteria listed in Table 1. In addition, if more than one valid primer shared a given 3' position, all but the shortest was automatically discarded as redundant. We employed two separate tests for primer-primer interaction, one based on a standard local alignment to detect stretches of complementary sequence, the other based on the worst-case ΔG of the 3'-tail of one primer interacting somewhere along the strand of another primer. These interaction criteria were used for screening individual primers as well as forward-reverse primer pairs, and for determining compatibility of two SNPs within a single multiplex PCR assay. Primer selection stringency is obviously a critical factor impacting the performance of any multiplex PCR design process. Fewer primers more carefully chosen may be more likely to produce a working assay but could undermine one's ability to identify high-multiplex designs. We have attempted to select primers based on a number of commonly employed selection criteria, recognizing that our overall performance would be directly impacted by any particular selection criteria.

**Table 1:** Primer design selection criteria. These criteria are used, where applicable, for determining the compatibility of forward and reverse primers within a given locus and for pair-wise compatibility between primers for different loci.

**Parameter**	**Allowed Range**
Length	17 – 24 bases
% GC	35 – 65%
T_m _(nearest neighbor)	57.0 – 70.0 C
T_m _Difference	3.0 C (for both candidate primer pairs and for all primers within a particular multiplex tube)
Base repeats	≤ 3 bases maximum
Product Size	60 – 200 bases
Distance to SNP	177 bases (5' end) 5 bases (3' end)
Self complementarity local alignment score	≤ 8.0 (match = 1.0, mismatch = 1.0, gap = -2.0)
3'-Tail alignment ΔG	≥ -4.5 kCal/mol

Using the above primer selection criteria, we generated an average of 1555.8 +/- 1249.4 primer pair candidates per SNP. Twenty-two of our 1,200 SNPs (1.8%) produced no valid primer candidates. For each SNP we randomly selected a primer pair and constructed the corresponding compatibility graph, where edges connect two compatible SNPs. The graph density was 0.299 +/- 0.005 over 10 random trials. As noted earlier, a random graph model is an imprecise representation for the multiplex PCR primer design problem because primer pair candidates are not independent, and because the primers for SNPs within a tube tend to become dominated by one of four non-interacting base pair combinations (G-T, G-A, C-T, or C-A.)

To further understand how SNP-pair compatibility probabilities depend on primer selection criteria, we randomized these criteria and evaluated the resulting graph density for 50 SNPs randomly chosen from human chromosome 21. While certain oligo-specific parameters were kept fixed, pair-wise compatibility criteria were chosen at random within certain specified ranges. The specific compatibility criteria we considered were:

• *Complementary Sequence Local Alignment Score*: Allowed to vary between +4 and +10 (assuming base match = +1, mismatch = -1, gap = -2). Typically, a threshold of +8.0 is used. A smaller scoring threshold is more stringent as we are disallowing primer pairs with less complementary sequence.

• *3' Tail ΔG Alignment Score*: This score measures the worst-case alignment of the 3' tail of one primer along any other part of another sequence. Current practice suggests that it is the 3' tail that is most critical to ensuring proper primer ligation. We allowed this threshold to range from 0.0 to -9.0 kCal/mol. (-4.0 to -6.0 kCal / mol is probably reasonable, although this scoring method is not widely used.) A higher (less negative) cutoff is more stringent as we are disallowing less-energetically favorable interactions.

• *Melting Temperature (Tm) Range*: The allowed melting temperature between any two primer pairs. A Tm difference of 3.0 degrees K is fairly standard. Here we allow the Tm difference to vary between 0.5 and 7.5 degrees K. (A smaller Tm difference is more stringent.)

**Figure 5:**
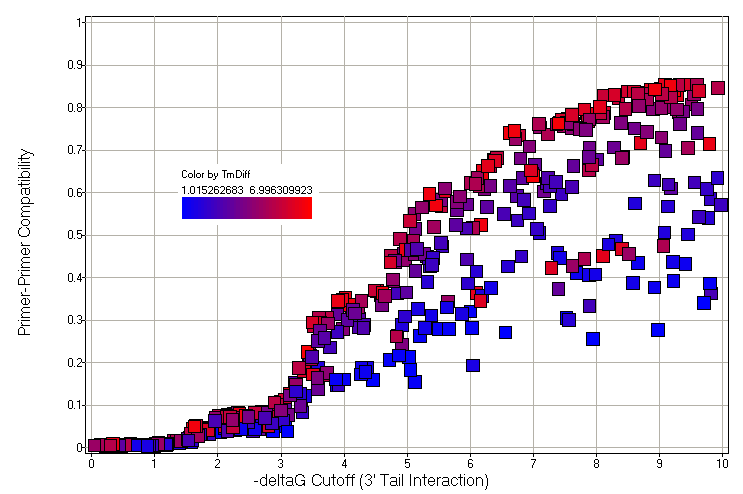
**Primer-primer compatibility probability and primer selection stringency. **This figure shows how a number of primer selection criteria impact the overall probability that two primers will be mutually compatible. If compatible primers are connected by edges in a graph, the resulting probability is equivalent to the graph density. This figure plots graph density as a function of 3' ΔG interaction. Each point represents a single trial where additional primer compatibility thresholds were randomly chosen within specified ranges. Considered were the 3' tail ΔG interaction, complementary sequence local alignment score, and melting temperature (Tm) difference. Points that are more red allow for high Tm differences while points that are more blue require smaller Tm differences. The impact of local alignment score thresholds, while not shown explicitly, is indirectly revealed by the multiple tiers (bands) across the graph, the lowest corresponding to score = +4 and the highest to score = +6 to +10.

The random selection of thresholds and the resulting generation of valid primers constituted a single trial. Within each trial, we randomly selected one primer pair for each locus and computed the resulting density of a graph where nodes represent particular SNPs having an assigned primer pair, and edges connect two multiplex-compatible SNPs. For two SNPs to be mutually compatible, all four primers must be pair-wise compatible using the selected thresholds. These thresholds are thus used to screen both intra- and inter-SNP primer pairs. The graph density was computed as the average of 10 densities each resulting from 10 random primer selection rounds. Figure 5 shows the relationship between graph density and the 3' tail interaction threshold.

Each point in the graph represents a particular trial. Red points have a high Tm difference threshold while blue points have a low Tm difference threshold. As expected, the resulting compatibility graph density increases as we loosen the constraint on the interaction ΔG. For a given ΔG cutoff, tighter constraints on the Tm difference will naturally tend to reduced SNP pair compatibility. The multiple performance tiers (clearly seen as multiple red bands across the chart) reflect different cutoffs for the standard complementary sequence local alignment score, the lowest being for Score = 4, while at the top, scores of 6–10 blend together in this figure. At ΔG = -4.5 kCal/mol, we expect graph densities no greater than about 30%. In other words, the probability that two SNPs, each assigned random primer pairs, are multiplex compatible is only about 30%.

### Algorithms

#### The fixed-assignment best-fit algorithm

We define a benchmark best-fit strategy for designing uniform M-plexes as follows:

Let G_N _be a multi-node graph with **N **nodes (SNPs).

Let M be the desired clique size (multiplexing level).

Let **T**= [T_1_, T_2_, ..., T_N/M_] cliques (tubes), initially empty.

Let **CANTASSIGN **be a set of unassignable nodes, initially empty.

1. Choose a node, **u**∊**N **and **u**∉**CANTASSIGN **at random.

2. Assign a random state (primer pair) to the node

3. Find all cliques in **T **that are compatible with **u**. (A clique is compatible if the size of the clique is less than M, and every node in the clique has an edge connected to **u **according to the appropriate edge matrices. Thus, if **u **is added to the clique, then it is still a clique. If there are one or more compatible cliques, we assign **u **to the *largest *clique, otherwise, we leave u unassigned.

4. For all unassigned nodes, choose a different state, ensuring that no state is chosen more than once. If a particular unassigned node **u **has no such state, then we add **u **to **CANTASSIGN**.

5. Repeat steps 1–4 until there are no more nodes to choose from. The resulting set of cliques, **T**, define our final solution. We measure coverage as a percentage of the nodes assigned to full tubes.

For N nodes (SNPs) and S states (primer pair candidates) per node, pre-computing and storing all possible primer-pair interactions in a multi-node graph would require **O**(N^2^S^2^) time and space. Therefore, when applying the above algorithm to real-world SNPs with potentially hundreds of candidate primer pairs (states) per SNP (node), we determine primer interactions (edge interactions) as needed in order to test a particular SNP for tube compatibility.

Step 3 provides our definition of "best-fit." The best tube is defined as the *largest *compatible tube. The rational for this strategy is that while each tube assignment reduces our probability of finding another assignment to the same tube, the reduction in probability is minimized when we make an assignment to the tube that is currently the largest. It should be noted, however, that in practice very little change in the performance of the algorithm is observed if we assign nodes to a random compatible tube, to the smallest compatible tube, or even to the first compatible tube. This is due to the fact that the probability of assigning a particular SNP to a particular tube decreases exponentially as the size of the tube (clique) grows larger. For sufficiently large cliques, it is unlikely that a SNP with a particular assigned primer set will be compatible with *any *tube. Most often, when a compatible tube is found, it is the only *compatible *tube among the  available choices, and thus there is little or no resulting difference in how we actually choose our tube.

#### Flexible-assignment best-fit

As noted above, we expect the probability of finding a compatible tube for a particular SNP in a particular state to decrease exponentially as the tube size increases. Suppose in attempting to assign a SNP to a particular tube, we find that it is incompatible with one other SNP which was originally assigned when the tube was small. It is relatively easy to find a compatible tube when they contain few SNPs because there are fewer interactions to consider. By contrast, if we find a nearly-compatible SNP when the tube is large, we should attempt to accommodate the SNP with the idea that low-probability SNP/Tube assignments are relatively rare and should thus be maintained whenever possible.

In general, suppose we have a tube containing k SNPs (a clique with k nodes). We test a SNP for compatibility with the tube and find that d ≤ k SNPs are incompatible with the SNP. Suppose furthermore that these d SNPs were assigned to the tube when the tube was of size k_1_, k_2_, ... k_d_. We claim that it is valuable to substitute our test SNP for the d incompatible SNPs whenever the total probability of assigning the d incompatible SNPs is greater than the probability of assigning the test SNP to a tube that excludes these d SNPs. That is:



If there are multiple tubes satisfying the above condition, we assign the SNP to the tube where  is maximized. In assessing these assignment probabilities, additional consideration could be given to the number of candidate primer pairs maintained by each SNP, as it is less likely that we will identify a compatible tube for SNPs with relatively few candidates with the idea that we should be more reluctant to remove such SNPs once a compatible assignment is determined. In this version, however, we considered only the tube sizes at the time each SNP is assigned. In the Fixed-Assignment Best-Fit algorithm, the size of each tube is monotonically increasing. Thus, if a SNP with a given primer candidate is not compatible with any tube, the primer pair candidate can be removed from further consideration and furthermore, if no primer candidate is compatible for any tube, then the SNP can be discarded from further consider. By contrast, the Flexible-Assignment Best-Fit algorithm requires that we reconsider the compatibility of SNPs and their associated primers within any modified tube. We thus specifically track which candidates have been tested on which tubes, and we update this status each time a particular tube is modified.

It is clear that the Flexible Assignment Best-Fit algorithm will eventually terminate with increasingly high probability because every SNP substitution we perform within a particular tube is assessed as having strictly lower probability than previous assignments.

## Authors' contributions

John Rachlin and Simon Kasif are responsible for the overall algorithmic design and theoretical analysis presented in this paper. John Rachlin and Simon Kasif introduced the framework of multi-node graphs in consultation with Noga Alon. John Rachlin implemented the algorithms and produced the initial results for both simulated and real human SNP data. John Rachlin discovered the phase transitions during the analysis of the simulated data which were explained by a theoretical analysis developed initially by Noga Alon and Vera Asodi and expanded and reported in [23]. Charles Cantor proposed the problem and provided the overall direction and oversight of the project. Chunming Ding was involved in the overall biotechnology design and also helped identify the representative primer selection criteria used as the basis for the optimization criteria used by the system. All authors contributed to outlining, writing and editing the manuscript with John Rachlin carrying the lion share of the write-up.
